# 2457. Improved Identification of Primary Infection Sites in Suspect Central Line Bloodstream Infections (CLABSI) through Preliminary Blood Culture Notifications

**DOI:** 10.1093/ofid/ofad500.2075

**Published:** 2023-11-27

**Authors:** Werner Bischoff, Cheryl Kieta, Tiffany LaFontaine, Corrianne Billings

**Affiliations:** Wake Forest University School of Medicine, Winston Salem, NC; Atrium Health Wake Forest Baptist, Winston Salem, North Carolina; Atrium Health Wake Forest Baptist, Winston Salem, North Carolina; Atrium Health Wake Forest Baptist, Winston Salem, North Carolina

## Abstract

**Background:**

Central Line Bloodstream Infections (CLABSI) pose a significant risk for patients with high burden of morbidity and mortality. Accurate and timely identification of these events is paramount for the development of effective interventions.

**Methods:**

A quasi-experimental (before/after) study included all inpatients with central venous catheters (CVC) admitted to five acute care hospitals in North Carolina. Starting in 03/2022 all preliminary blood culture results were evaluated within 24 hours by surveillance infection preventionists (SIP) and results indicative of potential CVC infection were escalated to medical teams. Escalation included identification of potential primary site infections, and discussion of the need for further testing and documentation. Mid-P exact tests were used to compare CLABSI burden before and after the intervention.

**Results:**

From 03/22 through 03/23 preliminary blood culture results in a total of 498 cases were reviewed by SIP. Of those, 201 (40%) were escalated to the IP team with 94 determined to be CLABSI, and 107 (53%) referred to further assessment by IP and medical teams. A primary infection site other than CVC was established based on escalation conversations in 23 events leading to a reduction in potential CLABSI cases by 11.4%. Standardized Infection Ratios for all and publicly reportable locations were reduced by 13% and 27% (table 1).

Of the 23 events interventions led to the establishment of a primary site of infection or documentation for exclusion criteria: pneumonia (9, 39%), burn (6, 26%), endocarditis (2, 9%), patient line injection (2, 9%), central line documentation clarification (1, 4%), intraabdominal infection (1, 4%), necrotizing enterocolitis (1, 4%), and skin infection (1, 4%). Interventions included imaging studies, culture collection of purulent sites, and documentation clarification including: abnormal/changes in wound characteristics, patient line injection, central line documentation.

**Figure:**
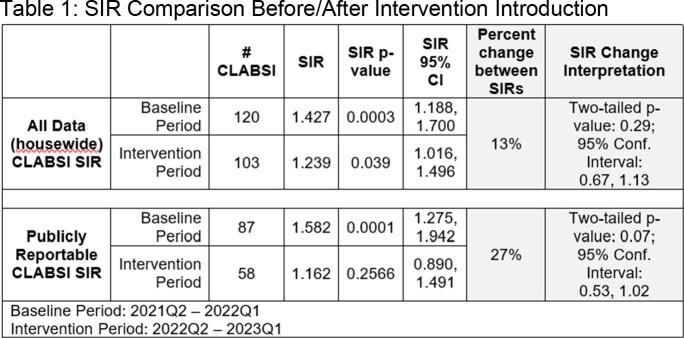

**Conclusion:**

Moving the process of CLABSI identification from finalized blood cultures to preliminary blood culture results improved the accuracy of CLABSI detection. Early inclusion of medical teams in the discussion of potential CLABSI increased awareness of the source of infection.

**Disclosures:**

**All Authors**: No reported disclosures

